# Prognostic Value of the Systemic Immune-Inflammation Index in Advanced Endometrial Cancer Treated with Pembrolizumab–Lenvatinib: A Retrospective Real-World Study

**DOI:** 10.3390/curroncol33080441

**Published:** 2026-07-23

**Authors:** Eleonora Palluzzi, Amedeo Cefaliello, Olga Martelli, Mariagrazia Distefano, Carolina Maria Sassu, Valentina Weger, Serena Maria Boccia, Laura Vertechy, Giorgia Russo, Luca Romano, Giacomo Corrado, Carolina Bottoni, Diana Giannarelli, Christian Marth, Anna Fagotti, Francesco Fanfani, Claudia Marchetti

**Affiliations:** 1 Dipartimento Scienze della Salute della Donna e del Bambino, Fondazione Policlinico Universitario Agostino Gemelli, IRCCS, 00168 Rome, Italylaura.vertechy@policlinicogemelli.it (L.V.);; 2 Dipartimento Scienze della Vita e Sanità Pubblica, Università Cattolica del Sacro Cuore, 00168 Rome, Italyluca.romano@specializzandi.unipg.it (L.R.); 3 Unit of Medical Oncology, Santa Maria della Misericordia Hospital, 06156 Perugia, Italy; 4 Facility of Epidemiology and Biostatistics—GSTeP, Fondazione Policlinico Universitario Agostino Gemelli, IRCCS, 00168 Rome, Italy; 5 Department of Obstetrics and Gynecology, Innsbruck Medical University, 6020 Innsbruck, Austria

**Keywords:** systemic inflammation index, endometrial cancer, pembrolizumab, lenvatinib

## Abstract

Background: The prognostic role of the Systemic Immune-Inflammation Index (SII) in patients with endometrial cancer (EC) treated with pembrolizumab plus lenvatinib (Len-Pem) is unknown. In this study, the association between baseline SII and progression-free survival (PFS) was evaluated. Methods: This is a monocentric, observational, retrospective/ambispective study including patients with advanced, recurrent, or metastatic mismatch repair proficient (pMMR) EC treated with Len-Pem. Results: Fifty-three patients were enrolled, with a median age of 64 years. Median PFS in the overall population was 7.1 months; median OS was 15 months. Patients with baseline SII ≤ 540 had significantly longer PFS (12.5 months) compared to those with SII > 540 (5.5 months; HR 2.94, *p* = 0.004). Higher SII was also significantly associated with increased risk of specific toxicities, including nausea, anorexia, and vomiting. The Overall Response Rate (ORR) was 42%, and Disease Control Rate (DCR) was 71%. Conclusion: Our real-world study supports the hypothesis-generation of a prognostic role for SII in predicting outcomes and toxicity in EC patients treated with Len-Pem. SII may serve as a cost-effective, accessible biomarker to stratify patients and hypothetically guide clinical decision-making. Further prospective studies are warranted to validate its clinical utility and to optimize dosing strategies that balance efficacy and tolerability.

## Simple Summary

The prognostic role of the Systemic Immune-Inflammation Index (SII) in endometrial cancer (EC) patients treated with immunotherapy remains unknown. We evaluated the association between baseline SII and progression-free survival (PFS). In this monocentric, observational, retrospective/ambispective study, we enrolled 53 patients with advanced, recurrent, or metastatic mismatch repair proficient (pMMR) endometrial cancer treated with pembrolizumab–lenvatinib (Len-Pem). Patients with baseline SII < 540 had significantly longer PFS (12.5 months) compared to those with SII ≥ 540 (5.5 months; HR 2.94, *p* = 0.004). Higher SII was also significantly associated with increased risk of specific toxicities, including nausea, anorexia, and vomiting. Our real-world study suggests the prognostic role of SII in predicting outcomes and toxicity in EC patients treated with Len-Pem.

## 1. Introduction

Endometrial cancer (EC) is the most common gynecological cancer in high-income countries, and its incidence is rising globally [1], predominantly affecting postmenopausal women. While most cases are diagnosed at an early stage and generally carry a favorable prognosis, approximately 15–20% of patients present with advanced or recurrent disease, which is associated with poor clinical outcomes and limited treatment options [1]. Patients with primary advanced and recurrent endometrial cancer are considered together in this setting because they share a similar therapeutic pathway, prognosis, and treatment indication once disease becomes unresectable or metastatic. Cytotoxic chemotherapy and radiotherapy offered limited efficacy in these settings, highlighting the need for novel therapeutic strategies [2,3].

Immune Checkpoint Inhibitors (ICIs) are widely applied for the treatment of various types of malignant tumors and demonstrate clinical benefits for selected patients [4]. ICIs such as pembrolizumab and dostarlimab have demonstrated remarkable clinical activity, particularly in tumors with microsatellite instability-high (MSI-H) or mismatch repair deficiency (dMMR), which are characterized by high mutational burden and increased immunogenicity.

However, the majority of endometrial carcinomas are mismatch repair proficient (pMMR) or microsatellite stable (MSS), and these tumors demonstrate limited responsiveness to ICIs monotherapy. The combination of pembrolizumab with lenvatinib, a multitargeted tyrosine kinase inhibitor (TKI), represents a significant advancement in the management of pMMR EC [5].

Despite its clinical efficacy, the combination of Len-Pem at the recommended dose was associated with significant treatment-related toxicity [5]. Additionally, treatment interruptions occurred in 70.2% of patients, and lenvatinib dose reductions in 62.9%, with only 8.9% of patients remaining on lenvatinib for ≥6 months without any dose reductions [6]. Therefore, there is a significant unmet clinical need to evaluate reliable biomarkers to predict the toxicity and efficacy of ICIs therapy and to promote individualized treatment regimens for patients who are candidates for immunotherapy.

The efficacy of ICIs may be modulated by several factors such as nutritional status [7], body mass index [8], PD-L1 expression [9], tumor mutation burden, or tumor-infiltrating lymphocytes [10]. The Systemic Immune-Inflammation (SII) index is an inflammation-related indicator, and it is calculated based on the peripheral lymphocyte, neutrophil, and platelet counts. This integrated parameter has been considered a clinically informative index that reflects the balance of inflammatory and immune status in cancer patients [11]. SII has been investigated as a marker to predict cancer survival in hepatocellular carcinoma [12]. With regard to lung cancer, the prognostic value of SII was reported in several studies, and most of them showed that the SII index could be a biological prognostic biomarker for lung cancer [13,14]. Similar data have also been reported in vulvar cancer [15]. Overall, the relationship between SII and survival outcomes in patients treated with ICIs remains uncertain. Few data about SII are available for endometrial cancer, but the role of this inflammatory marker for immunotherapy has not been studied so far [16,17,18].

We therefore hypothesized that baseline SII could be associated with clinical outcomes and treatment-related toxicity in patients with advanced or recurrent pMMR endometrial cancer treated with Len-Pem.

The aim of this study was to evaluate the prognostic value of baseline SII and its changes during treatment in a real-world cohort of patients receiving Len-Pem.

## 2. Materials and Methods

This is an observational, retrospective/ambispective, monocentric study that included patients with advanced, recurrent or metastatic EC treated with Lenvatinib-Pembrolizumab combination according to the label. Patients with primary advanced disease and those with recurrent disease were included because both groups represented the target population eligible for pembrolizumab plus lenvatinib in routine clinical practice. Consistent with the KEYNOTE-775 study design, patient selection was based on treatment indication rather than the timing of disease presentation [5]. Baseline clinicopathological characteristics, treatment exposure, radiological assessments, and toxicity data were collected retrospectively through review of medical records. Survival status and follow-up information were subsequently updated at the time of data lock (April 2025), allowing prospective collection of outcome data for patients who were still alive and under active follow-up.

The inclusion criteria were as follows: histologically confirmed endometrial carcinoma; advanced, recurrent, or metastatic disease not amenable to curative treatment; mismatch repair proficient (pMMR) or microsatellite stable (MSS) status; at least one cycle of Len-Pem.

The exclusion criteria included the following: participation in a concurrent clinical trial; active infections or uncontrolled comorbidities; prior treatment with immune checkpoint inhibitors.

Demographic and clinical data were collected at initial diagnosis and at recurrence and recorded in an electronic database (Microsoft Excel) for analysis.

The primary objective of the study was to assess the association between the baseline SII and clinical outcomes, focusing on progression-free survival (PFS). SII was calculated as = (neutrophils count × platelets count)/lymphocyte count.

Secondary objectives included evaluating correlation between SII and overall survival (OS) and toxicities. We also describe the efficacy and safety of Len-Pem in the intention-to-treat (ITT) population and incidence of adverse events (AEs), as well as treatment discontinuations and dose reductions due to AEs. In addition, data on post-progression treatments were collected and analyzed in all patients who experienced a progression during or after Len-Pem.

This study was approved by the local institutional ethics committee, and all participants provided written informed consent in accordance with the Declaration of Helsinki.

According to standard clinical practice, the recommended starting dose was pembrolizumab 200 mg administered intravenously every 3 weeks, combined with lenvatinib 20 mg orally once daily. Treatment parameters including the number of administered cycles, starting and final maintenance doses of lenvatinib, as well as any dose interruptions, reductions, or permanent discontinuations, were recorded. Adverse events were recorded according to Common Terminology Criteria for Adverse Events (CTCAE) version 5.1. Tumor response and disease progression were assessed according to the Response Evaluation Criteria in Solid Tumors (RECIST) version 1.1 [19].

PFS was defined as the time interval from the starting date of Len-Pem until disease progression (defined as objective radiological disease progression using modified RECIST version 1.1 or clinical progression) or last contact or death. Patients who did not experience disease progression were censored at the date of the last follow-up visit. OS was defined from the time between the starting date of Len-Pem treatment to death or the last follow up visit for living patients. Disease Control Rate (DCR) was defined as the proportion of patients who achieve tumor response (partial response (PR) or complete response (CR) or stable disease (SD)).

For SII calculation, complete blood counts (CBCs) performed before the first and last cycle of pembrolizumab–lenvatinib treatment were retrospectively retrieved. CBCs were not performed at standardized intervals with respect to radiological assessment of disease.

Baseline SII values were available for 42 of the 53 enrolled patients. Missing SII values were mainly attributable to the absence of complete blood count measurements within the predefined baseline assessment, in particular due to the absence of the lymphocyte value, not routinely reported in clinical records. No imputation procedures were performed because of the ambispective nature of the study and the limited sample size.

Analyses were conducted on available cases only. Therefore, all findings should be interpreted as exploratory and hypothesis-generating.

Kaplan–Meier and log-rank analyzes were used to evaluate PFS while, once the assumption of proportionality of risks had been verified, a multivariable Cox proportional hazard model was used to analyze SII for its prognostic relevance. The optimal cut-off values of SII were determined by Receiver Operating Characteristic (ROC) curves. Median follow-up was calculated according to the inverse Kaplan–Meier method; *p*-value for toxicity was calculated with the Mann–Whitney non-parametric test. The ΔSII was calculated using the non-parametric Wilcoxon Signed Rank Test to evaluate the association with PFS and OS distribution, while the Mann–Whitney test was used to evaluate the ΔSII between patients who discontinued for progression of disease (PD) and those who discontinued for toxicity.

## 3. Results

Fifty-three EC patients were enrolled at our institution and treated with Len-Pem combination from November 2022, with a data cut-off date in April 2024. Patients’ characteristics at baseline are shown in Table 1.

The median age at time of diagnosis was 64 years (range 48–78). All the patients enrolled had proficient mismatch repair proteins (pMMR) status evaluated by an immunohistochemical method. The most common histologic subtypes were serous (38%), endometrioid (28%), and mixed (21%), followed by other histotypes (13%).

Twenty-six (49%) patients were treated with previous pelvic radiotherapy. Twenty-three (43%) had previously received vaginal brachytherapy. Only one patient underwent brachytherapy without pelvic radiotherapy. Most patients (89%) had received two lines of platinum-based chemotherapy before Len-Pem. Adjuvant chemotherapy was counted as one line of therapy. Fourteen patients (26%) had pre-existing autoimmune disease (Table S1).

The sites of disease were distributed as follows: 45% visceral, 34% lymph-node, 32% peritoneal and 2% other sites of disease. Most patients (74%) had single sites of disease. The median number of administered cycles was seven (range 1–23).

Fifty-two (98%) patients were evaluable for response, clinical outcomes and toxicities. One patient was not evaluable because only one treatment cycle was administered due to clinical deterioration.

At data cut-off analysis, 30 patients (57%) were still alive, 22 (42%) had died and 1 (2%) was lost at follow-up. Of the evaluable patients, 33 (64%) discontinued treatment for PD and eight (15%) for treatment limiting toxicity. Eleven patients (21%) were still undergoing treatment. Of these, two (15%) had oligoprogression involving only one of either one disease site, one pulmonary and one bone lesion, and they were treated with stereotactic body radiotherapy (SBRT) continuing Len-Pem. No association was found for ΔSII between patients who discontinued for PD and those who discontinued for toxicity (*p* = 0.62) (Figure S1). Six (11%) out of all the patients started with a lower dose of lenvatinib due to clinical conditions. In five of these patients, the dose was further reduced, and, in two, treatment was definitively suspended. A lenvatinib dose reduction was necessary in 42 (79%) patients who started with the standard lenvatinib dose: in 19 patients (40%), to 14 mg; in 21 patients (44%), to 10 mg; and, in two patients (5%), to 8 mg (Table 2).

The objective response rate (ORR) was 42% (95% CI: 29–56) including 38% partial response and 4% complete response. The disease control rate was 71% (95% CI: 59–83) (Table 3).

After a median follow-up of 15.0 months (range: 2–33), median PFS was 7.1 months (95% CI: 4.3–9.9) in the overall population. For patients with endometrioid histology (29%) median PFS was 14.3 months (CI: 4.8–23.8); for those with serous histology (38%), 8.8 months (CI: 0–20.4); and, for those with mixed histology (23%), 6.1 months (CI: 5.7–6.5), respectively. Median OS was 15 months (2–33). No significant difference in terms of OS was observed in this population. Given the limited number of patients in each histologic subgroup, these findings should be interpreted as exploratory.

**Figure 1:**
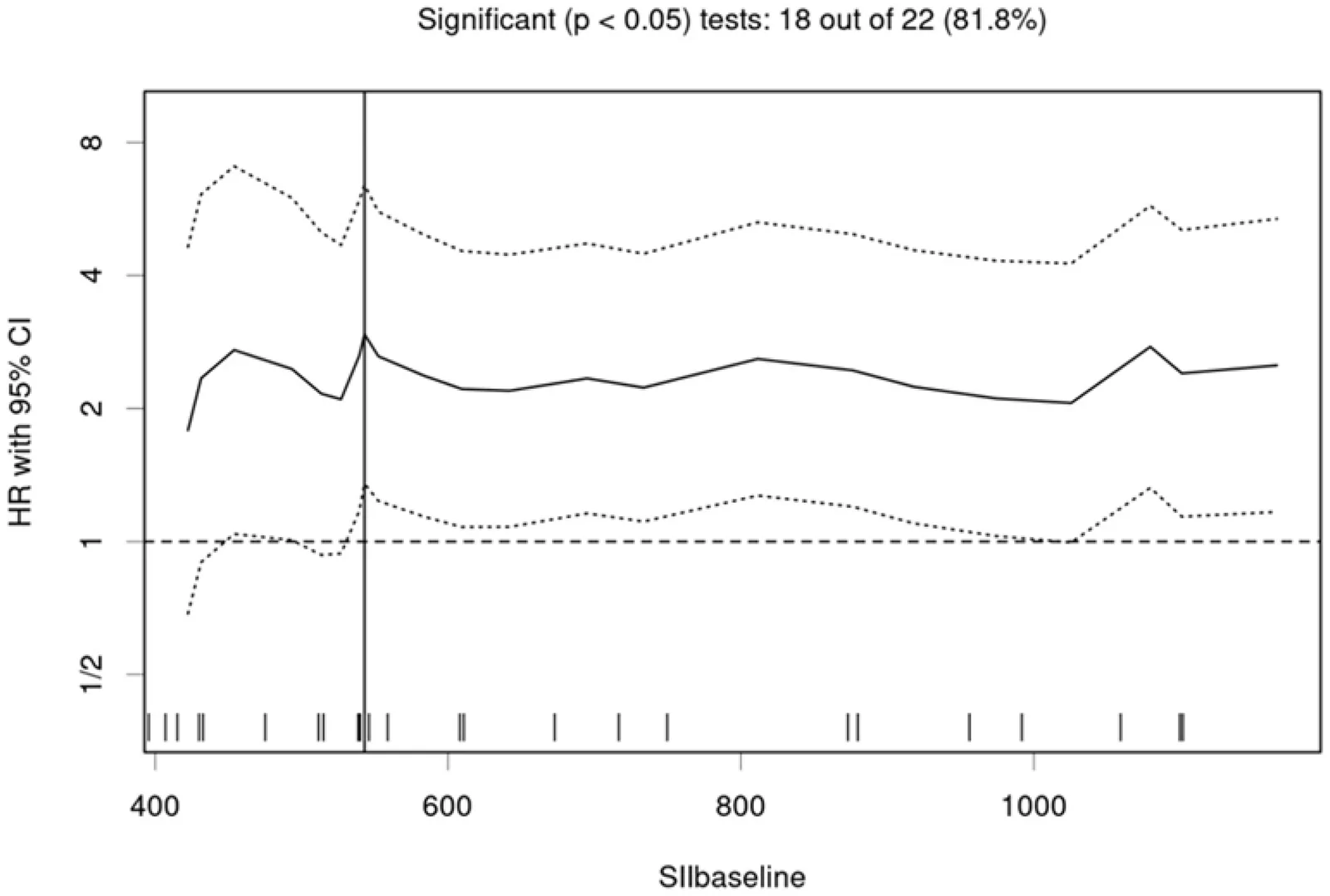
Baseline SII cut-off analysis for progression-free survival. Hazard ratio estimates for progression-free survival according to tested baseline Systemic Immune-Inflammation Index (SII) cut-off values. The ROC-derived exploratory cut-off of 540 showed the highest discriminatory performance within this cohort and was subsequently used to stratify patients into low-SII and high-SII groups. Abbreviations: SII, Systemic Immune-Inflammation Index; HR, hazard ratio.

In 42 (79%) patients, SII was available at baseline. The median value of baseline SII was 1009 (SD = 917). Baseline SII was associated with PFS: the hazard ratio, for each 100-unit increase in SII, was 1.08 (95% CI: 1.03–1.13), *p* < 0.001. We analyzed all possible cut-offs of SII in relation to the hazard ratio through Receiver Operating Characteristic (ROC) curves (Figure S2). Eighteen cut offs were significant, and the optimal cut-off identified in our cohort to discriminate prognosis was 540 (Figure 1).

**Figure 2:**
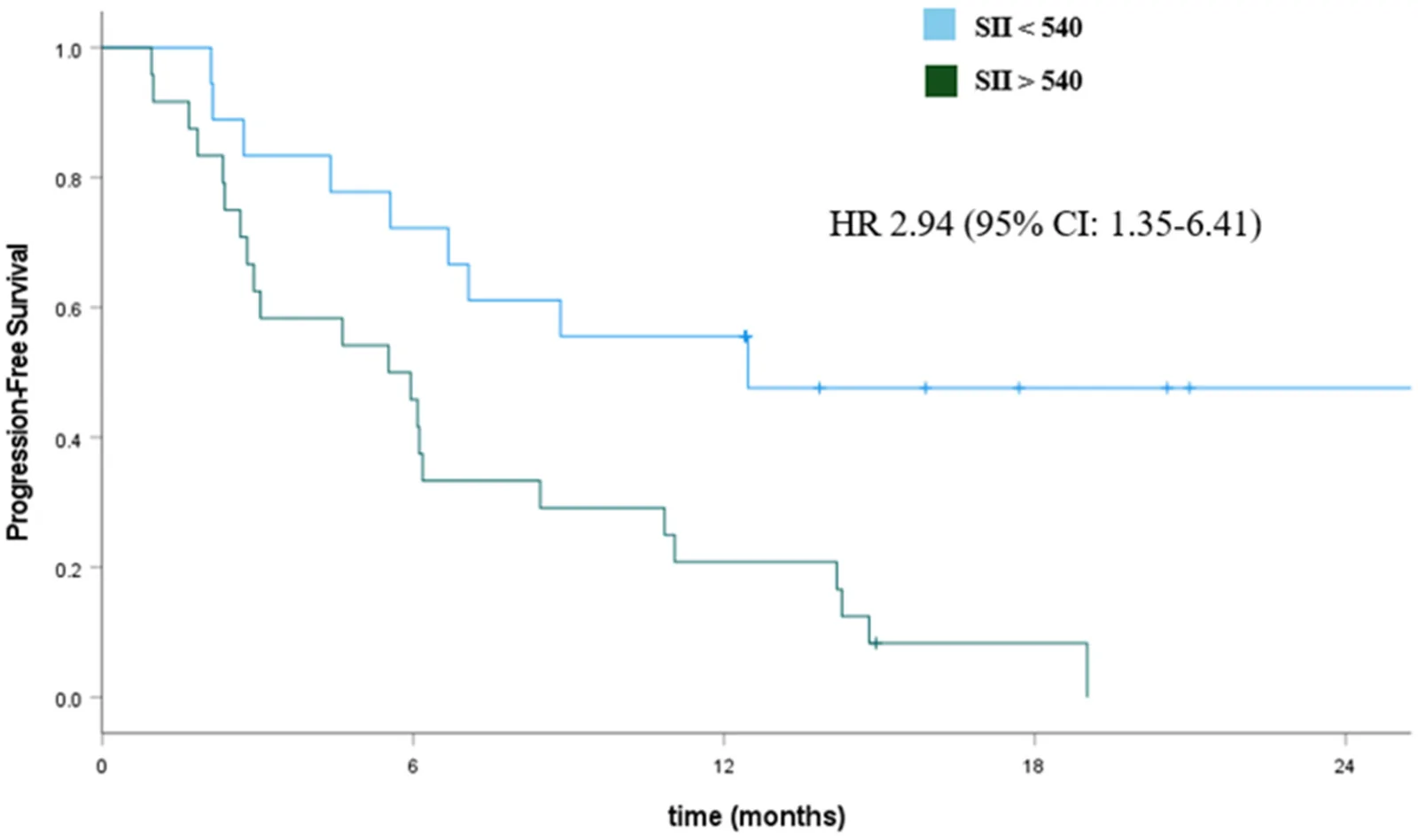
Kaplan–Meier analysis of progression-free survival according to baseline SII. Patients with baseline SII ≤ 540 had longer median PFS compared with patients with SII > 540 (12.5 vs. 5.5 months; HR 2.94 (95% CI: 1.35–6.41; *p* = 0.004).

Eighteen patients had baseline SII ≤ 540; 24 patients had SII > 540 (Table 4 and Table S2). The mPFS was 12.5 months (95% CI: NE) for patients with baseline SII ≤ 540 and 5.5 months for patients with SII baseline > 540, HR 2.94 (95% CI: 1.35–6.41), *p* = 0.004 (Figure 2). ORR was 44% for patients with baseline SII ≤ 540, while it was 30% for patients with baseline SII > 540 (Table 3). No association was found between ΔSII with PSF and OS (*p* = 0.48 for PFS and *p* = 0.43 for OS).

Subsequent systemic treatment after progression on Len-Pem was administered in 30 patients. The most frequently administered regimen was platinum-based chemotherapy (27%) followed by pegylated liposomal doxorubicin (23%), weekly paclitaxel (20%), clinical trial (17%), oral cyclophosphamide (7%) and gemcitabine (7%), respectively.

The most frequent adverse events of any grade were fatigue (58%), hypertension (50%), hypothyroidism (48%), arthralgia (35%), diarrhea (21%), nausea (21%) and anemia (21%), followed by proteinuria (13%), anorexia and constipation (12%), vomiting (10%) and weight reduction (6%).

Patients who experienced vomiting, anorexia, or nausea exhibited higher median SII values than those with other toxicities, at 1605.4 (*p* = 0.006), 2273.4 (*p* = 0.036) and 2008.3 (*p* = 0.016), respectively (Table 4).

SII data at the end of treatment were available for thirty-seven patients. Median SII at the end of treatment was 1374.5 (75–1555). There was no statistically significant difference between baseline and end of treatment SII value (*p* = 0.57).

We constructed a model including SII, histology, prior radiotherapy and disease burden. SII is the only significant factor for the univariate analysis and remains significant also in the multivariate analysis (Table S3).

## 4. Discussion

This real-world observational study supports the prognostic significance of SII in advanced or recurrent endometrial cancer (EC) patients treated with Len-Pem. Our data demonstrate a statistically significant association between baseline SII and progression-free survival (PFS), with higher SII levels correlating with poorer outcomes. Specifically, patients with SII > 540 exhibited a markedly shorter median PFS (5.5 months) compared to those with SII ≤ 540 (12.5 months), with a hazard ratio of 2.94. This observation suggests that SII may serve as a readily accessible biomarker for early stratification of patients in terms of likely benefit from immunotherapy. We also observed significant associations between higher SII and certain adverse events (AEs), including nausea, anorexia, and vomiting, suggesting a possible link between systemic inflammation and treatment-related toxicities. However, based on the present data, high baseline SII should not yet influence treatment selection in these patients due to the small sample and the retrospective nature of the study. Our findings should be regarded as the generation of a hypothesis and not prospectively validated data.

Our study adds novel insights by exploring the hypothetical prognostic utility of SII in this therapeutic context. SII has been validated as a prognostic biomarker in several malignancies, including hepatocellular carcinoma [12], lung and gastric cancers, and one study evaluated this issue in prostate cancer [20,21,22]. In lung cancer, higher SII values were associated with poorer outcomes in patients treated with immune checkpoint inhibitors (ICIs), reinforcing the concept that systemic inflammation may counteract immunotherapeutic efficacy [23]. Beyond its antiangiogenic activity, lenvatinib has been shown to modulate the tumor microenvironment by reducing VEGF-mediated immunosuppression and promoting immune-cell infiltration, thereby potentially enhancing the activity of immune checkpoint blockade. Given the lower intrinsic immunogenicity of pMMR endometrial cancers compared with dMMR tumors, host-related inflammatory factors may play an important role in determining treatment outcomes in this setting [5].

Few studies have evaluated the prognostic role of SII in endometrial cancer (EC), and none has specifically assessed its value in patients receiving immunotherapy. A recent meta-analysis demonstrated that elevated pretreatment SII was significantly associated with worse overall survival (OS) and progression-free survival (PFS) in EC; however, all the included studies were conducted in non-immunotherapy settings, leaving its prognostic significance during immune checkpoint inhibition largely unexplored [18]. Hu et al. further reported that SII was significantly associated with cancer recurrence, with higher SII values predominantly observed in patients who experienced disease recurrence, suggesting a potential role for this biomarker in risk stratification [24].

An important consideration is the substantial heterogeneity in SII cut-off values reported across the gynecologic oncology literature. Previous studies have identified markedly different thresholds, depending on disease setting and patient population. Matsubara et al. [17] reported cut-off values ranging from approximately 910 to 931 in endometrial cancer, whereas Lin et al. [25] identified a threshold of approximately 494 in patients receiving neoadjuvant chemotherapy. Similarly, studies evaluating immunotherapy in cervical cancer have reported cut-off values exceeding 1100 [26]. The meta-analysis by Ji et al. [18] confirmed the prognostic relevance of SII in endometrial cancer while also highlighting considerable inter-study heterogeneity. For example, studies evaluating surgically treated endometrial cancer, neoadjuvant chemotherapy cohorts, and patients receiving immunotherapy-based regimens may capture distinct biological and clinical contexts, leading to different optimal threshold values. Consequently, the identification of a single universally applicable SII cut-off remains challenging. This variability represents a major obstacle to clinical implementation and underscores the need for prospective validation and methodological standardization before SII can be routinely incorporated into clinical decision-making. Therefore, rather than focusing on a specific numerical threshold, future research may benefit from evaluating whether the prognostic value of SII remains consistent across different patient populations and treatment settings. Such an approach may be more informative than attempting to define a universally applicable cut-off value.

Our data extend these findings by showing an association between SII and survival outcomes specifically in pMMR EC patients receiving Len-Pem, suggesting that systemic inflammation may be a marker of resistance to immune-modulatory therapy. Cancer-related inflammation is considered a key mechanism in tumor progression, and tumor-induced inflammatory responses can cause corresponding changes in peripheral neutrophil, lymphocyte, and platelet count. Considering that SII is a systemic marker of host immune function, the association of SII and PFS might be explained by reduced response to immunotherapy in patients with an inflamed, possibly exhausted, immune status. A low SII value may reflect a reduction of the neutrophil and platelet counts and an increase in lymphocyte count, potentially indicating the favorable condition of the immune system.

Interestingly, nearly half of the patients in our cohort had received prior pelvic radiotherapy, a factor that could have influenced baseline SII. Radiotherapy has well-documented immunomodulatory effects, including alterations in systemic cytokine levels, bone marrow suppression, and potential lymphopenia [26,27,28]. Although not directly evaluated in this study, it is plausible that prior radiation contributed to higher SII levels via long-term myelosuppressive effects, especially lymphopenia, thus altering the inflammatory index.

The rationale for evaluating ΔSII was based on the hypothesis that dynamic changes in systemic inflammation during treatment may better reflect evolving host–tumor interactions than a single baseline measurement. Although no significant association was observed in our cohort, longitudinal inflammatory biomarkers remain an area of interest and should be investigated in larger prospective datasets.

Regarding treatment efficacy, our real-world population achieved a median PFS of 7.1 months and an ORR of 42%, consistent with the KEYNOTE-775 trial (median PFS 7.2 months, ORR 31.9%). Subgroup analysis revealed that patients with pMMR endometrioid histology had notably longer PFS (14.3 months), suggesting histological subtype plays a substantial role in treatment responsiveness. In our study, disease control rate (DCR) (71%) is consistent with prior findings, supporting the external validity of trial data despite differences in population characteristics and treatment management.

In our cohort, 79% of patients required dose reductions, with some initiating treatment at a reduced dose due to clinical fragility. While the KEYNOTE-775 trial did not stratify survival outcomes by dose reduction, emerging real-world data and retrospective analyses suggest that early lenvatinib dose reductions may not compromise effectiveness if they allow for prolonged treatment exposure and better tolerance. However, we observed a non-significant but numerically shorter PFS in patients requiring early dose modification, warranting further investigation into optimal dosing strategies balancing efficacy and safety.

The safety profile observed in this cohort is consistent with known adverse event patterns of Len-Pem combination therapy. Notably, toxicities were significant in the population, with fatigue, hypertension, and hypothyroidism being the most frequent. Vomiting, nausea, and anorexia may be associated with higher baseline SII, suggesting that the Systemic Inflammation Index may also predict susceptibility to adverse events. Although elevated baseline SII was associated with an increased incidence of selected gastrointestinal toxicities, these findings should be interpreted cautiously. Multiple adverse events were evaluated simultaneously, and no formal correction for multiple comparisons was applied. Consequently, some of the observed associations may reflect chance findings related to multiple testing. Therefore, these results should be considered exploratory and hypothesis-generating and require confirmation in larger independent cohorts.

Among our patients, 26% had pre-existing autoimmune diseases. These patients are traditionally excluded from registration trials due to concerns about immune-related adverse events (irAEs). While our sample is limited, no excess immune-related toxicity was observed in this subgroup, consistent with emerging literature suggesting that pre-existing autoimmune conditions do not necessarily contraindicate ICIs therapy, provided careful monitoring is employed [29].

This study has several limitations. The retrospective, single-center design and relatively small sample size limit the generalizability of our findings and increase the risk of overfitting. Indeed, it is not possible to determine whether SII is a prognostic biomarker reflecting overall disease biology or a predictive biomarker specific to Len-Pem benefit. Furthermore, baseline SII values were unavailable for approximately 21% of the enrolled patients. Although the missing data were mainly related to the absence of complete laboratory assessments during the baseline period, the possibility of selection bias in the survival analysis cannot be excluded. Given the limited sample size and corresponding events-per-variable ratio, the estimated hazard ratios may be subject to statistical instability and reduced precision. Therefore, the multivariable model should be interpreted as exploratory and hypothesis-generating rather than confirmatory, and the observed associations require validation in larger independent cohorts.

Another limitation is the lack of standardization of CBC with respect to radiological reassessments, which did not allow us to perform a correlation between SII and PD.

Although we explored other cut-offs reported in the literature, none showed significant correlation with prognosis in our cohort [11,12,13]. Furthermore, potential confounders, such as comorbidities and prior treatments, were not uniformly controlled or adjusted in the multivariate models.

An additional limitation relates to the identification of the optimal SII threshold. The threshold of 540 was identified through optimization procedures performed within the same dataset subsequently used for survival analyses. While this approach is frequently adopted in exploratory biomarker studies, it may increase the risk of overfitting and multiplicity bias, potentially leading to inflation of the observed effect size and overestimation of the discriminatory performance of the biomarker. Furthermore, the absence of an independent validation cohort prevents assessment of the reproducibility and generalizability of the proposed threshold. Consequently, the identified cut-off should be regarded as exploratory and requires external validation before any potential clinical application.

Our analyses suggest that SII may serve as a readily accessible biomarker identifying patients with a poor outcome and limited benefit from immunotherapy.

Patients with high SII could potentially benefit from closer monitoring, early dose adjustments, or alternative treatment strategies. Furthermore, the association between SII and certain toxicities highlights the potential of SII as a potential marker of treatment tolerability, which could help guide clinical decision-making and optimize supportive care.

Importantly, the present study was not designed to assess whether SII is predictive of benefit from pembrolizumab–lenvatinib. Demonstration of predictive value would require an appropriate comparator cohort and assessment of differential treatment effects according to SII status.

Future research should focus on validating these findings in larger, multicentric prospective cohorts and exploring the integration of SII with other immune-related markers, such as PD-L1 expression, tumor-infiltrating lymphocytes, or genomic signatures. Investigating whether interventions that modulate systemic inflammation (e.g., anti-inflammatory agents, nutritional optimization) can enhance the efficacy of ICIs in high-SII patients also represents an area of clinical interest. Furthermore, longitudinal assessment of SII during treatment may help clarify whether dynamic changes in systemic inflammation reflect treatment response, emerging resistance, toxicity risk, or disease progression. Such investigations may ultimately define the most appropriate role of SII within future biomarker-based risk stratification strategies in advanced endometrial cancer.

## 5. Conclusions

In conclusion, our findings support the hypothesis that SII may represent an accessible prognostic biomarker for the early stratification of patients with pMMR EC treated with Len-Pem and highlight the challenges of maintaining dose intensity in real-world settings. However, given the retrospective design, limited sample size, and absence of external validation, these findings should be considered exploratory and hypothesis-generating. Consequently, the present findings should not be interpreted as supporting immediate clinical implementation of SII-guided treatment strategies. Future prospective studies are warranted to validate the prognostic significance of SII and to determine its potential role within future biomarker-based risk stratification strategies while preserving efficacy and minimizing toxicity.

## Figures and Tables

**Table 1:** Demographic and disease characteristics of the patients at baseline.

**Age**	
Median (range) ≤65 yr 65 < yr > 75 ≥75 yr	64 (48–78) yr 30 (57) 22 (41) 1 (2)
**ECOG Performance Status Score**	**No. (%)**
0 1 2	40 (75) 13 (25) -
**Mismatch repair status**	**No. (%)**
pMMR dMMR	53 (100) -
**FIGO stage at diagnosis**	**No. (%)**
I–II III–IV NA	18 (34) 32 (60) 3 (6)
**Histologic Type**	**No. (%)**
Endometrioid Serous Clear cell Squamous Mixed histology Not specified Carcinosarcoma	15 (28) 20 (38) 1 (2) 1 (2) 11 (21) 4 (7) 1 (2)
**p53 status**	**No. (%)**
p53 mutated p53 wild-type NA	20 (37) 27 (50) 6 (13)
**ER/PgR status**	**No. (%)**
−/− +/+ +/− −/+ NA	8 (15) 26 (49) 7 (14) 0 (0) 12 (22)
**Previous pelvic radiotherapy**	**No. (%)**
Yes No	26 (49) 27 (51)
**Previous brachytherapy**	**No. (%)**
Yes No	23 (43) 30 (57)
**Previous lines of platinum-based chemotherapy**	**No. %**
1 >1	6 (11) 47 (89)
**Previous hormonal therapy**	**No. %**
Yes No	5 (9) 48 (91)
**Pre-existing autoimmune disease**	**No. %**
Yes No	14 (26) 39 (74)
**Site of disease**	**No. (%)**
Visceral disease Lymph nodes Peritoneal Other sites	24 (45) 18 (34) 17 (32) 1 (2)
**Single site vs. multiple site**	**No. (%)**
*Single sit* Visceral single site Lymph nodes Peritoneal Other site *Multiple site*	39 (74) 15 (28) 11 (21) 12 (23) 1 (2) 14 (26)

**Table 2:** Lenvatinib starting dose, dose reductions, and permanent discontinuation.

Lenvatinib Treatment	N° (%)
**Initial dose** - 20 mg - 14 mg	47 (89) 6 (11)
**Reduction LEN dose (initial dose 20 mg) ** - One-step reduction Two-step reduction Three-step reduction	19 (40) 21 (44) 2 (5)
**Reduction LEN dose (initial dose 14 mg) ** - One-step reduction - Two-step reduction	4 (66) 1 (17)
**Definitive suspension** - Initial dose 20 mg - Initial dose 14 mg	0 (0) 2 (3)

Abbreviations: LEN, lenvatinib.

**Table 3:** Activity and efficacy of pembrolizumab–lenvatinib treatment.

	All Patients	SII > 540	SII ≤ 540	NA	*p*-Value (SII ≤ 540 vs. SII > 540)
**No. of total patients**	53	24	18	11	
Median number of cycles (range)	7 (1–23)				
No. of evaluable patients	52	23	18	11	
Objective Response Partial Response Complete Response	20 2	7 0	8 0	5 2	
Stable Disease	15	8	6	1	
Progressive Disease	13	7	3	3	
Pseudoprogression	2	1	1	0	
Objective response rate	42%	30%	44%	63%	0.35
Disease control rate	71%	65%	78%	72%	0.38

Abbreviations: SII, Systemic Immune-Inflammation Index; NA, not available.

**Table 4:** Safety report and correlation between median SII and toxicities.

Adverse Event (AE)	N. of Patients Without AE	Median SII of Patients Without AE	N. of Patients with AE	Median SII of Patients with AE	*p*-Value
Hypertension	16	1314.3	26	701.3	0.19
Hypothyroidism	17	971.1	25	910.1	0.90
Diarrhea	31	937.9	11	926.0	0.27
Nausea	31	696.9	11	1605.4	0.016
Inappetence	36	755.9	6	2008.3	0.036
Vomiting	37	753.9	5	2273.4	0.006
Weight loss	39	921.1	3	1112.7	0.58
Fatigue	12	881.7	30	956.1	0.67
Arthralgia	24	1176.1	18	613.1	0.026
Proteinuria	35	948.4	7	867.0	0.77
Anemia	31	786.5	11	1352.7	0.29
Constipation	36	818.4	6	1633.5	0.45

## Data Availability

The data presented in this study are available on request from the corresponding author.

## Supplementary Materials

The following supporting information can be downloaded at: https://www.mdpi.com/article/10.3390/curroncol33080441/s1, Table S1. Demographic and disease characteristics of the patients with a previous autoimmune disease according to SII baseline; Table S2. Patients’ features according to SII < 540 and SII > 540; Table S3. Univariate and multivariate analysis according to SII, previous RT, histotype and burden of disease; Figure S1. ΔSII between patients who discontinued for PD and those who discontinued for toxicity; Figure S2. Receiver Operating Characteristic (ROC) curves for all possible cut offs of SII.

## Abbreviations

SII	Systemic Immune-Inflammation Index
EC	Endometrial Cancer
Len-Pem	Pembrolizumab–Lenvatinib
pMMR	Mismatch Repair Proficient
PFS	Progression-Free Survival
ORR	Overall Response Rate
DCR	Disease Control Rate
ICIs	Immune Checkpoint Inhibitors
MSI-H	Microsatellite instability-high
dMMR	Mismatch repair deficiency
MSS	Microsatellite stable
ITT	Intention to treat
AEs	Adverse events
CTCAE	Common Terminology Criteria for Adverse events
RECIST	Response evaluation criteria in solid tumors
PR	Partial Response
CR	Complete Response
SD	Stable Disease
CBC	Complete blood counts
OS	Overall Survival
PD	Progression of disease
SBRT	Stereotactic body radiotherapy
HR	Hazard Ratio
irAEs	Immune-related adverse events
