# Supplementary material for: Prognostic Value of the Systemic Immune-Inflammation Index in Advanced Endometrial Cancer Treated with Pembrolizumab–Lenvatinib: A Retrospective Real-World Study
Source: Curr Oncol. 2026 Jul 23;33(8):441. doi: 10.3390/curroncol33080441 (PMC13510687; doi:10.3390/curroncol33080441)
Supplement: Supplementary file 1 [file curroncol-33-00441-s001.zip › curroncol-4324616-supplementary.pdf]

Table S1. Demographic and disease characteristics of the patients with a previous autoimmune disease according to SII baseline.

	SII		
	≤ 540 (3)	>540 (8)	NA (3)
**Autoimmune disease**			
• Autoimmune thyroiditis	3 (100)	6 (76)	3 (100)
• Rheumatoid arthritis	0 (0)	1 (12)	0 (0)
• Angioedema	0 (0)	1 (12)	0 (0)
**Median Age (years)**	63	58,5	63
**mPFS (months)**	12	6	3
**p53 status n (%)**			
• P53 mutated	2 (66)	3 (38)	1 (34)
• P53 wild-type	1 (34)	5 (62)	2 (66)
**ER/PgR status n (%)**			
• -/-	0 (0)	2 (25)	0 (0)
• +/+	1 (34)	4 (50)	3 100
• +/-	2 (66)	1 (12)	0 (0)
• -/+	0 (0)	0 (0)	0 (0)
• Non disponibile	0 (0)	1(12)	0 (0)
**Histology n (%)**			
• Endometrioid	1 (33)	2 (25)	1 (33)
• Mixed histology	1 (33)	3 (37)	1 (33)
• Serous	1 (33)	3 (37)	1 (33)
• Other	0 (0)	0 (0)	0 (0)
**Previous radiotherapy n (%)**			
• Yes	0 (0)	5 (62)	2 (66)
• No	3 (100)	3 (38)	1 (34)
**Previous brachytherapy n (%)**			
• Yes	1 (34)	5 (62)	1 (34)
• No	2 (66)	3 (38)	2 (66)
**Previous lines n (%)**			
• 1	1 (33)	0 (0)	0 (0)
• 2	1 (33)	7 (87)	2 (66)
• ≥ 3	1 (33)	1 (13)	1 (34)
**Site of disease n (%)**			
• Distant metastasis	3 (100)	4 (50)	2 (66)
• Pelvic recurrence	0 (0)	0 (0)	0 (0)
• Distant metastasis + Pelvic recurrence	0 (0)	4 (50)	1 (34)
**Site of metastasis n (%)**			
• Visceral	1 (33)	1 (12)	2 (66)
• Lymph nodes disease	1 (33)	1 (12)	0 (0)
• Peritoneal	1 (33)	4 (50)	1 (34)
• Other	0 (0)	2 (25)	0 (0)
**mNeutrophils (x10³/mm3)**	2.1	3.81	NA
**mPlatelets (x10³/mm3)**	196	317	NA
**mLymphocytes (x10³/mm3)**	1.53	1.15	NA
**mSII baseline**	335	1181	NA
**Hypotyroidism n (%)**			
• Yes	2 (66)	6 (75)	1 (34)
• No	1 (34)	2 (25)	2 (66)
**Hypertension n (%)**			
• Yes	2 (66)	3 (38)	3 (100)

• No	1 (34)	5 (62)	0 (0)
**Diarrhea n (%)**			
• Yes	1 (34)	2 (25)	0 (0)
• No	2 (66)	6 (75)	3 (100)
**Fatigue n (%)**			
• Yes	3 (100)	5 (62)	2 (66)
• No	0 (0)	3 (38)	1 (33)
**Artrhalgia n (%)**			
• Yes	3 (100)	5 (62)	1 (34)
• No	0 (0)	3 (38)	2 (66)
**Anemia n (%)**			
• Yes	2 (66)	4 (50)	2 (66)
• No	1 (34)	4 (50)	1 (34)
**Neutropenia n (%)**			
• Yes	0 (0)	1 (13)	0 (0)
• No	3 (100)	7 (87)	3 (100)

This supplementary table summarizes the demographic, clinical, pathological, and treatment-related characteristics of patients with pre-existing autoimmune diseases treated with pembrolizumab plus lenvatinib. Patients were stratified according to baseline SII status using the exploratory ROC-derived cut-off of 540, while a separate category is reported for patients with unavailable baseline SII values. Variables include autoimmune disease subtype, age, progression-free survival, molecular profile, histology, previous treatments, disease distribution, inflammatory blood-cell counts, and treatment-related toxicities. Owing to the limited number of patients included in this subgroup analysis, findings should be interpreted as descriptive and exploratory. Abbreviations: SII, Systemic Immune-Inflammation Index; PFS, progression-free survival; ER, estrogen receptor; PgR, progesterone receptor; NA, not available.

Table S2. Patients’ features according to SII < 540 and SII>540.

	**SII**	
	**≤ 540**	**>540**
**Median age (years)**	64	66
**Median PFS (months)**	12.5	5.5
**Molecular risk n (%)**		
• POLE mutated	0 (0)	0 (0)
• p53 mutated	7 (39)	11 (46)
• NMSP	8 (44)	9 (37)
• NA	3 (17)	4 (17)
**Histology n (%)**		
• Endometrioid	4 (22)	7 (29)
• Mixed	5 (28)	5 (21)
• Serous	6 (33)	9 (37)
• Others	3 (17)	3 (13)
**Previous radiotherapy n (%)**		
• Yes	6 (34)	15 (62)
• No	12 (66)	9 (38)
**Previous line of chemotherapy n (%)**		
• 1	2 (11)	1 (4)
• 2	16 (89)	21 (88)
• ≥ 3	0 (0)	2 (8)
**Site of disease n (%)**		
• Distant metastases	15 (83)	16 (67)
• Pelvic recurrence	0 (0)	1 (4)
• Distant metastastases and pelvic recurrence	3 (17)	7 (29)
**Sites of metastasis**		
• Visceral	8 (40)	7 (24)

• Lymph nodes	8 (40)	7 (24)
• Peritoneal	4 (20)	12 (41)
• Other sites	0 (0)	3 (11)
**m Neutrophils count(x10³/mm3)**	3.3	3.64
**m Platelets count (x10³/mm3)**	210.5	276.5
**m Lymphocytes count (x10³/mm3)**	1.88	0.89

Baseline demographic, clinical, pathological, and treatment-related characteristics stratified according to the exploratory ROC-derived SII threshold of 540. This analysis was performed to assess the distribution of potential prognostic factors between SII groups and to identify possible sources of confounding in subsequent survival.

Table S3. Univariate and multivariate analysis according to SII, previous RT, Hystotype and burden of disease.

	**UNIVARIATE**		**MULTIVARIABLE**	
	**HR (95% CI)**	P	HR (95% CI)	P
**SII**				
<540	Ref.	0.007	Ref.	0.006
> 540	2.94 (1.35-6.41)		3.33 (1.41-7.88)	
**Previous RT**				
No	Ref.	0.43	Ref.	0.79
Yes	1.29 (0.68-2.43)		1.12 (0.47-2.66)	
**Hystotype**				
Endometrioid	0.68 (0.31-1.52)	0.64	0.44 (0.17-1.17)	0.23
Serous	0.84 (0.40-1.78)		0.60 (0.22-1.61)	
Other	Ref.		Ref.	
**Burden of disesase**				
Single site	Ref.	0.10	Ref.	0.13
Multiple sites	1.87 (0.89-3.97)		2.13 (0.80-5.71)	

Univariate and multivariable analyses evaluating the association between selected clinical variables and progression-free survival. Variables included in the multivariable model were selected on the basis of clinical relevance and results from univariate analyses. Owing to the limited sample size and number of events, the multivariable model should be interpreted as exploratory and hypothesis-generating rather than confirmatory.

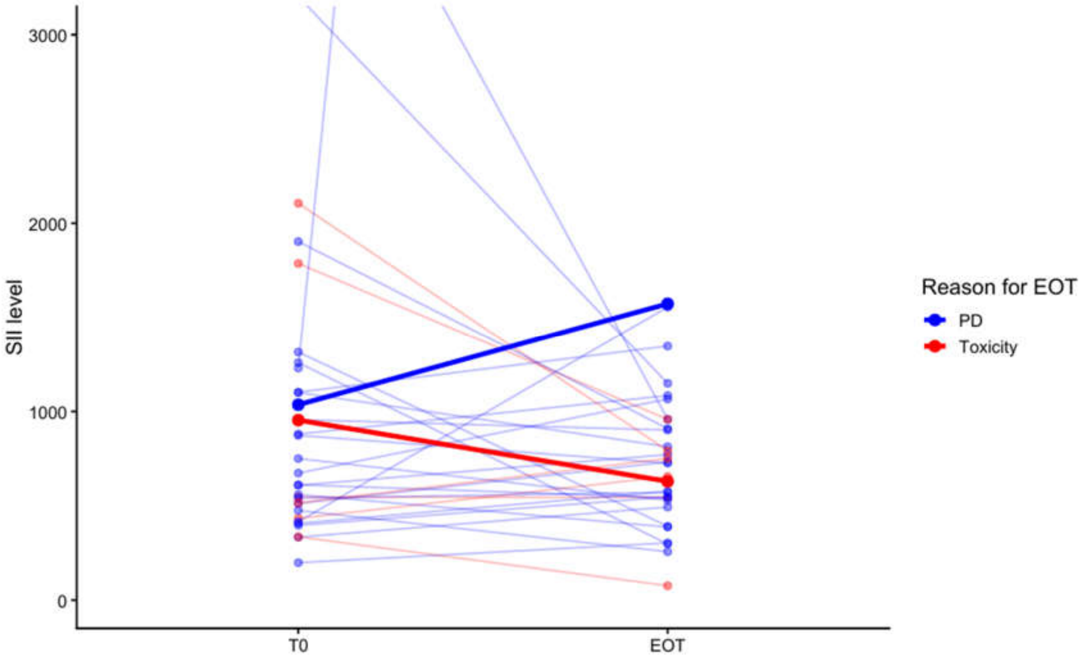

Figure S1. Longitudinal changes in Systemic Immune-Inflammation Index (ΔSII) according to reason for treatment discontinuation. Individual patient trajectories and median values of SII are shown between treatment initiation (T0) and end of treatment (EOT). Patients were grouped according to the reason for treatment discontinuation, namely disease progression (PD) or treatment-related toxicity. This exploratory analysis was performed to evaluate whether dynamic changes in systemic inflammatory status during treatment were associated with different clinical outcomes. No statistically significant association was observed between ΔSII and the reason for treatment discontinuation. Abbreviations: ΔSII, change in Systemic Immune-Inflammation Index; PD, progressive disease; EOT, end of treatment.

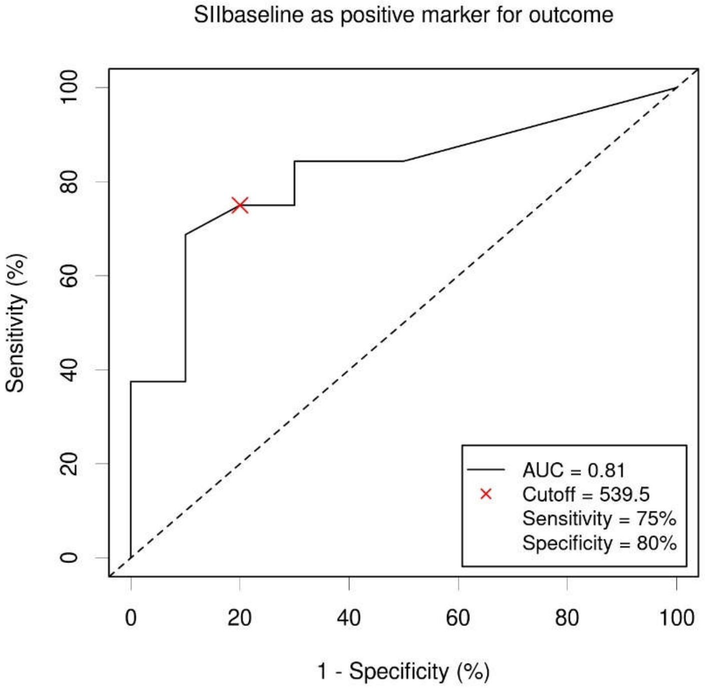

Figure S2. Receiver Operating Characteristic (ROC) curves for all possible cut offs of SII. ROC curve evaluating the ability of baseline Systemic Immune-Inflammation Index (SII) to discriminate progression-free survival outcomes. The exploratory cut-off value of 540 was selected according to the highest discriminatory performance observed within the study cohort. Because both cut-off derivation and outcome assessment were performed within the same dataset, the proposed threshold should be considered exploratory and requires external validation.
